# Prognostic value of MRI-derived masticator space involvement in IMRT-treated nasopharyngeal carcinoma patients

**DOI:** 10.1186/s13014-015-0513-6

**Published:** 2015-09-25

**Authors:** Youping Xiao, Jianji Pan, Yunbin Chen, Shaojun Lin, Ying Chen, Jingfeng Zong, Yanhong Fang, Qiaojuan Guo, Bijuan Chen, Linbo Tang

**Affiliations:** Department of Radiology, Fujian Provincial Cancer Hospital & Institute, No. 420, Fuma Road, Fuzhou, 350014 Fujian Province P. R. China; Department of Radiation Oncology, Fujian Provincial Cancer Hospital & Institute, No. 420, Fuma Road, Fuzhou, 350014 Fujian Province P. R. China; Provincial Clinical College of Fujian Medical University, No. 1, Xueyuan Road, Fuzhou, 350014 Fujian Province P. R. China

**Keywords:** Nasopharyngeal carcinoma, Masticator space, Magnetic resonance imaging, Prognosis, Staging category

## Abstract

**Objectives:**

This retrospective study reassessed nasopharyngeal carcinoma (NPC) patients treated with intensity-modulated radiation therapy (IMRT), to determine the significance how magnetic resonance imaging (MRI)-derived masticator space involvement (MSI) affected patients’ prognosis.

**Methods:**

One thousand one hundred ninety seven NPC patients who had complete set of MRI and medical records were enrolled. Basing on their MRI findings, the T-categories of tumors were identified according to the seventh edition of American Joint Committee on Cancer staging system, which considers MSI a prognostic indicator for NPCs. Rates of overall survival (OS), local relapse-free survival (LRFS), regional relapse-free survival (RRFS) and distant metastasis-free survival (DMFS) were analyzed by the Kaplan-Meier method, and the Log-Rank test compared their differences. Cox regression analysis was employed to evaluate various prognostic factors systematically. Statistical analyses were conducted with SPSS 18.0 software, P value < 0.05 was considered statistically significant.

**Results:**

Medial pterygoid muscle (MPM) was involved in 283 (23.64 %) cases, of which lateral pterygoid muscle (LPM) was concurrently affected in 181 (15.12 %) and infratemporal fossa (ITF) in 19 (1.59 %). Generally, MSI correlated with an OS, LRFS, and DMFS consistent with a T4-stage diagnosis (*P* > 0.05). Although different degrees of MSI presented a similar OS and DMFS (*P* > 0.1), tumors involving LPM had a relatively poorer LRFS than those affected the MPM only (*P* = 0.027), even for subgroup of patients composed of T3 and T4 classifications (*P* = 0.035). A tumor involving MPM brought an LRFS consistent with a T2 or T3-stage disease (*P* > 0.1). If the tumor affected LPM or ITF concurrently, the survival outcomes were more consistent with a T4-stage disease (*P* > 0.1). Nevertheless, compared to tumor infiltrating MPM, those invading LPM or ITF more frequently spread into other concurrent sites that earned higher T-staging categories. Moreover, multivariate analyses indicated the degree of MSI was a significant prognostic factor for the OS of NPCs (*P* = 0.036).

**Conclusions:**

Degree of MSI is a significant prognosticator for the OS of IMRT-treated NPCs, and the prognosis of patients with lateral MSI extension (LPM and ITF) were shown to be significantly worse than those affected only MPM or the T3-stage disease. Thus, it is highly recommended that lateral MSI extension be a higher T-staging category.

## Background

Nasopharyngeal carcinoma (NPC) arises from nasopharyngeal mucosa, and presents an invasive biological behavior that is prone to infiltrate the surrounding structures, especially those lying laterally in parapharyngeal space, masticator space (MS), and infratemporal fossa (ITF) [1]. NPC is known to be a radiosensitive tumor type, and chemoradiotherapy (CRT) is the primary treatment modality [2–4]. Reported 5-year overall survival (OS) rates for NPC patients have ranged from 58.6 to 83 %, and pretreatment tumor-node-metastasis (TNM) staging is used to guide assessments of the patient’s prognosis [5–8]. However, the T-categories and prognostic value of NPCs with a tumor involving MS are controversial [9–11].

MS is an intact anatomical space that contains the masticatory muscles, the posterior body and ramus of the mandible, and the mandibular division of the trigeminal nerve (V3). Reports of the frequency of masticator space involvement (MSI) in NPC have varied widely, ranging from 19.7 to 61 % [9–11]. Since most studies emphasized that MSI was one of the significant prognostic factors for NPC patients [9, 11], the American Joint Committee on Cancer (AJCC) staging system and the Chinese staging system that was revised in 2008 both recommended MSI as an independent factor for the T-stage classifications of NPC. However, a complicating matter is the fact that standards for T-staging categories, as set by the 2008 Chinese staging system, differed from those of AJCC [12]. Moreover, the AJCC changed its guidelines in the most recent edition (seventh edition) [13], and now considers the MS to be composed of a medial part (contains the medial pterygoid muscle [MPM] and lateral pterygoid muscle [LPM]) and a lateral part (contains the temporal and masseter muscle). This is a departure from the fifth and sixth editions that defined tumors with MSI as only affecting the lateral part of anatomical MS (also termed “infratemporal fossa [ITF]”), and categorized them as T4 disease. In the seventh edition, however, tumors involving the medial or lateral part (or both) are categorized as T4 disease [12, 13]. On the other hand, the 2008 Chinese staging system categorizes tumors involving the MPM only as T3 tumors, and those concurrently infiltrating other masticatory muscles are categorized as T4 tumors. Given these inconsistencies, it is imperative to build a consensus on the standards for T-staging categories of NPC with MSI. Besides, most previous studies assessed the prognosis of MSI in NPCs treated with conventional radiotherapy or mixed with intensity-modulated radiation therapy (IMRT). Nowadays, however, IMRT is extensively treated as the standard radiotherapy technique for NPC [14–16] and obtains an excellent local control rate, even for those with locally advanced diseases (i.e., T3 or T4 classifications) [8, 15]. Nevertheless, the prognostic value of MSI in NPCs treated with IMRT has not yet been accurately assessed.

Certain anatomical characteristics make it difficult to assess MSI through clinical examinations alone, so imaging modalities are essential to evaluate this region more efficiently [17–19]. Previously, computed tomography (CT) was valuable in the pretreatment TNM staging for NPC, especially in determining the T-staging categories. Compared to CT, whereas, magnetic resonance imaging (MRI) can provide a better tissue contrast and a multiplanar capability [18–20], which allows for a more accurate evaluation of the primary nasopharynx tumor’s extension into adjacent structures, in particular the MS or ITF. For these reasons, both the AJCC and the 2008 Chinese staging systems recommend MRI as the optimal imaging modality for T- and N-staging in newly diagnosed NPC [12, 13]. Accordingly, King et al. have demonstrated the more excellent diagnostic power of MRI with a sensitivity, specificity, and accuracy of 100, 93, and 95 %, respectively, when compared to endoscopy [20, 21]. Indeed, routine MRI with T2-weighted fast spin-echo and contrast-enhanced T1-weighted images in axial and coronal planes can clearly determine whether a tumor has spread into the MS and/or ITF [19].

Taking these advances of MRI into consideration, we retrospectively re-evaluated a cohort of consecutive newly diagnosed NPC patients who had been treated with IMRT and also had complete MRI images, medical records, and follow-up data in order to accurately assess whether MSI should influence the porgnoses and T-staging category of NPCs.

## Methods

### Ethics statements

This retrospective study was approved by the Institutional Review Board of Fujian Provincial Cancer Hospital & Institute (No. 200908). Permission was given to conduct this retrospective study without obtaining informed consent. All information was anonymized and de-identified prior to analysis.

### Patient selection and pretreatment evaluation

Data were collected from 1241 consecutive and newly diagnosed NPC patients who were clear of any distant organ metastasis. All patients had been resorted to and treated with IMRT at the Fujian Provincial Cancer Hospital & Institute between July 2005 and December 2011. Of the 1241 cases, 44 did not have a pretreatment MRI and so were excluded from this study. The remaining 1197 cases with complete MRI data were recruited, who were histopathologically confirmed as having NPC via analysis of a naso-pharyngoscopy biopsy. Based on their MRI findings, we staged all patients according to the criteria of the seventh edition of the AJCC staging system [13]. Table 1 summarized the clinical characteristics of enrolled patients.

**Table 1:** The characteristics, staging categories, and treatment regimens of NPC patients

Characteristics	All patients	MSI	MPM only	LPM	ITF
No. of patients	1197	283 (23.64 %)^a^	102 (8.52 %)	181 (15.12 %)	19 (1.59 %)
Male	905 (75.61 %)	224 (79.15 %)	77 (75.49 %)	147 (81.22 %)	14 (73.68 %)
Female	292 (24.39 %)	59 (20.85 %)	25 (24.51 %)	34 (18.78 %)	5 (26.32 %)
Median age (y)	46	46	46	46	46
Age range (y)	11-84	11-84	11-84	12-79	22-76
Pathological types^b^					
WHO type III	1134 (94.74 %)	270 (95.41 %)	97 (95.10 %)	173 (95.58 %)	16 (84.21 %)
WHO type II	51 (4.26 %)	8 (2.83 %)	2 (1.96 %)	6 (3.32 %)	3 (15.79 %)
WHO type I	12 (1.00 %)	5 (1.76 %)	3 (2.94 %)	2 (1.10 %)	0
Clinical Stages^c^					
Stage I	57 (4.76 %)	0	0	0	0
Stage II	314 (26.23 %)	11 (3.89 %)	6 (5.88 %)	5 (2.76 %)	0
Stage III	541 (45.20 %)	112 (39.57 %)	62 (60.79 %)	50 (27.62 %)	1 (5.26 %)
Stage IVa	223 (18.63 %)	137 (48.41 %)	30 (29.41 %)	107 (59.12 %)	16 (84.21 %)
Stage IVb	62 (5.18 %)	23 (8.13 %)	4 (3.92 %)	19 (10.50 %)	2 (10.53 %)
T classifications					
T1	295 (24.64 %)	0	0	0	0
T2	225 (18.8 %)	12 (4.24 %)	7 (6.86 %)	5 (2.76 %)	0
T3	441 (36.84 %)	120 (42.40 %)	64 (62.75 %)	56 (30.94 %)	0
T4	236 (19.72 %)	151 (53.36 %)	31 (30.39 %)	120 (66.30 %)	19 (100 %)
N classifications					
N0	170 (14.20 %)	50 (17.67 %)	22 (21.57 %)	28 (15.47 %)	6 (31.58 %)
N1	675 (56.39 %)	148 (52.30 %)	51 (50.00 %)	97 (53.59 %)	6 (31.58 %)
N2	290 (24.23 %)	62 (21.91 %)	25 (24.51 %)	37 (20.44 %)	5 (26.32 %)
N3a	26 (2.17 %)	8 (2.82 %)	0	8 (4.42 %)	0
N3b	36 (3.01 %)	15 (5.30 %)	4 (3.92 %)	11 (6.08 %)	2 (10.52 %)
Chemotherapy					
Induction	919 (76.78 %)	257 (90.81 %)	93 (91.18 %)	164 (90.61 %)	19 (100 %)
Concurrent	466 (38.93 %)	114 (40.28 %)	30 (29.41 %)	84 (46.41 %)	3 (15.79 %)
Adjuvant	493 (41.19 %)	125 (44.17 %)	37 (36.27 %)	88 (48.62 %)	11 (57.89 %)

NPC = Nasopharyngeal carcinoma; MSI = Masticator space Involvement; MPM = Medial pterygoid muscle; LPM = Lateral pterygoid muscle; ITF = infra temporal fossa

^a^Numbers in parentheses are percentages

^b^The 3rd edition of World Health Organization (WHO) classification of pathological feature in 2003

^c^Staging system and definitions of TNM classification were according to the seventh edition of the American Joint Committee on Cancer (AJCC) Classification [13]

### Treatment regimens

All patients were initially treated with definitive IMRT. The mean dose of radiotherapy was 70.35 Gy (range from 61.6 Gy to 86.65 Gy), and the mean fraction was 31.5 (range from 28 to 49) with a 2.25 Gy per fraction. A detailed description of the IMRT has been published previously [15, 22]. A total of 1029 patients (85.96 %) received platinum-based chemotherapy, as follows (see Table 1): induction in 253 (21.14 %), concurrent in 71 (5.93 %), adjuvant in 16 (1.34 %), induction-concurrent in 212 (17.71 %), induction-adjuvant in 294 (24.56 %), concurrent-adjuvant in 23 (1.92 %), and induction-concurrent-adjuvant in 160 (13.37 %). Whenever possible, patients who relapsed or had a persistent tumor after completing initial treatments received the salvage treatments of intracavitary brachytherapy, surgery, or further adjuvant chemotherapy.

### MR imaging

Head and neck MRI scans covering the area from the lower temporal lobe to the supraclavicular were conducted on all enrolled patients according to accepted protocols on a 1.5 T MR system (Signa Excite 1.5 T HD Twin Speed, GE Healthcare, WI, USA) or a 3.0 T MR system (Achieva 3.0 T, Philips Healthcare, Best, Netherlands). Before any anti-tumor treatments, all patients were subjected to five standard sequences as follow: 1) axial T1-weighted imaging-fast spin echo (T1WI-FSE) with the parameters of repetition time (TR)/echo time (TE) = 550/9.2 ms, field of view (FOV) = 36 cm × 36 cm, matrix = 160 × 160, slices = 36, thickness = 6 mm, gap = 0 mm, number of excitations (NEX) = 2; 2) axial proton density-weighted imaging (PDWI) or T2-weighted imaging with short TI inversion recovery (T2WI-STIR): TR/TE = 3200/70 ms, FOV = 36 cm × 36 cm, matrix = 960 × 960, slices = 36, thickness = 4 mm, gap = 0 mm, NEX = 4; 3) coronal T2WI-STIR: TR/TE = 2,327/63 ms, FOV = 36 cm × 36 cm, matrix = 160 × 160, slices = 18, thickness = 4 mm, gap = 0 mm, NEX = 2; 4) sagittal T1WI-FSE: TR/TE = 600/9.2 ms, FOV = 36 cm × 36 cm, matrix = 160 × 160, slices = 18, thickness = 6 mm, gap = 0 mm, NEX = 2; 5) post-contrast enhanced acquisition of axial T1WI-FSE with fat suppression (fs): TR/TE = 1215/9.2 ms, FOV = 36 × 36 mm, matrix = 160 × 160, slices = 36, thickness = 6 mm, gap = 0 mm, NEX = 2. In the post-contrast acquisition, a gadolinium-based agent (Gadopentetate dimeglumine, Magnevist; Bayer Healthcare, Berlin, Germany) was intravenously injected at a rate of 1.5 ml/sec with a dose of 0.1 mmol per kilogram of the patient’s body weight followed by a 20 ml saline flush. Among the sequences listed above, axial orientation was perpendicular to C3, and the coronal orientation was parallel to C3. The total time for collecting a conventional MR scan was about 15 to 20 min.

**Fig. 1:**
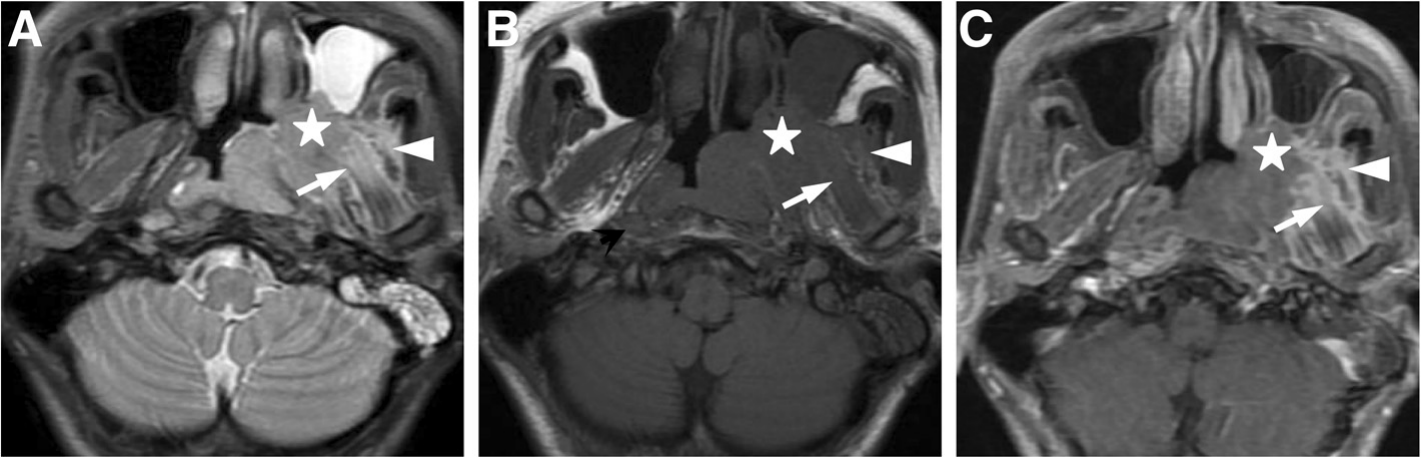
MR images of a 40-year old man with undifferentiated nonkeratinizing squamous cell nasopharyngeal carcinoma. MR images demonstrate NPC’s involvement of the MPM and LPM (long arrow), and the temporal muscle (arrowhead) as well as pterygopalatine fossa(star), which present a high signal intensity on axial PDWI image (**a**), a medium or low signal intensity on axial T1WI-TSE image (**b**), and an obvious enhancement on axial contrast-enhanced T1WI-TSE image with fat suppression (**c**). (MRI = Magnetic resonance imaging; NPC = Nasopharyngeal carcinoma; MPM = Medial pterygoid muscle; LPM = Lateral pterygoid muscle; PDWI = Proton density-weighted imaging; T1WI-TSE = T1 weighted imaging-Turbo spin echo)

All acquired MR images were reinterpreted independently by two radiologists specializing in head and neck MRI and who had eight and ten years of experience, respectively. Any disagreements were resolved by consensus. The diagnostic criterion for MSI was as follow (Fig. 1): primary tumor’s extension beyond the parapharyngeal space and pterygopalatine fossa and the erosion of the anatomical MS, which raises the signal intensity on the PDWI or T2WI-STIR images and significantly enhances the signal on the post-contrast T1WI-FSE images, both axial and coronal, in whole or in part and, at the same time, presents the erosion or disappearance of high signal intensity in the parapharyngeal space or pterygopalatine fossa on T1WI-FSE images.

### Follow-up and statistical analyses

The median follow-up time was 57 months (range from 7 to 105 months). Overall survival (OS) rate was calculated from the first day of diagnosis to the death date or the last follow-up, local relapse-free survival (LRFS) to the date of local relapse, regional relapse-free survival (RRFS) to the date of regional relapse, and distant metastasis-free survival (DMFS) to the date of distant metastasis, respectively. All statistical analyzes were conducted with the SPSS software (Version 18.0, Inc., Chicago, IL, USA). The frequency of different degrees of MSI among different T-staging categories was analyzed and compared by the Chi-square test. Survival curves were analyzed by the Kaplan-Meier method and differences compared by the Log-Rank test. Cox regression analysis was applied to evaluate the various prognostic factors systematically in NPC patients. A two-tailed P value < 0.05 was considered statistically significant.

## Results

### Patients with masticator space involvement

Using achieved MRI data, MSI were apparent in 283 cases (23.64 %), of which 102 (8.52 %) cases involved the MPM only, and the other 181 (15.12 %) infiltrated both the MPM and LPM. Furthermore, 19 (1.59 %) patients with LPM erosion also affected the ITF (including 19 temporal muscle and 5 masseter muscle). The characteristics, staging categories, and treatment regimens for NPC patients with different degrees of MSI were detailed in Table 1. Furthermore, a total of 271 (95.76 %) patients were treated with platinum-based chemotherapy, including induction in 73 (26.94 %), concurrent in 8 (2.95 %), adjuvant in 2 (0.74 %), induction-concurrent in 65 (23.98 %), induction-adjuvant in 82 (30.26 %), concurrent-adjuvant in 4 (1.48 %), and induction-concurrent-adjuvant in 37 (13.65 %).

### The survival outcomes of patients with masticator space involvement

For the entire group of 1197 patients, the 5-year OS, LRFS, and DMFS rates were 81.7, 93.4, and 83.2 %, respectively. Patients developed local recurrence, regional recurrence, and distant metastasis in 79 (6.6 %), 52 (4.3 %), and 20 (16.8 %) cases, respectively. Only 20 (1.67 %) patients presented with both local and regional recurrence, and 219 (18.3 %) ultimately died of the disease. As for patients with MSI, the 5-year OS, LRFS, and DMFS rates were 71.7, 90.5, and 77.7 %, respectively, and local recurrence, regional recurrence and distant metastasis developed in 27 (9.5 %), 11 (3.9 %) and 63 (22.3 %) cases, respectively. And there were 80 (28.3 %) who died by the ultimate follow-up, incluiding 61 distant metastasis or local-regional recurrences and 19 treatment-related complications. Furthermore, by comparing the survival outcomes between different degrees of MSI (only MPM, LPM, and ITF), the 5-year OS rates were 77.5, 68.5, and 63.16 %, LRFS rates were 95.1, 87.8, and 84.21 %, RRFS rates were 97.1, 95.6, and 89.47 %, and DMFS rates were 80.4, 76.2, and 63.16 %, respectively. And their corresponding local recurrence developed in 5/102 (4.9 %), 22/181 (12.2 %), and 3/19 (36.84 %) cases, respectively, regional recurrence developed in 3/102 (2.9 %), 8/181 (4.4 %), and 2/19 (10.53 %), respectively, and distant metastasis developed in 20/102 (19.6 %), 43/181 (23.8 %), and 7/19 (36.84 %), respectively. Interestingly, there were only 12 patients identified as MSI alone (without extension to any T3 or T4 structures), and their OS, LRFS, RRFS, and DMFS were much higher, at 83.3, 100, 100, and 91.7 %, respectively.

**Fig. 2:**
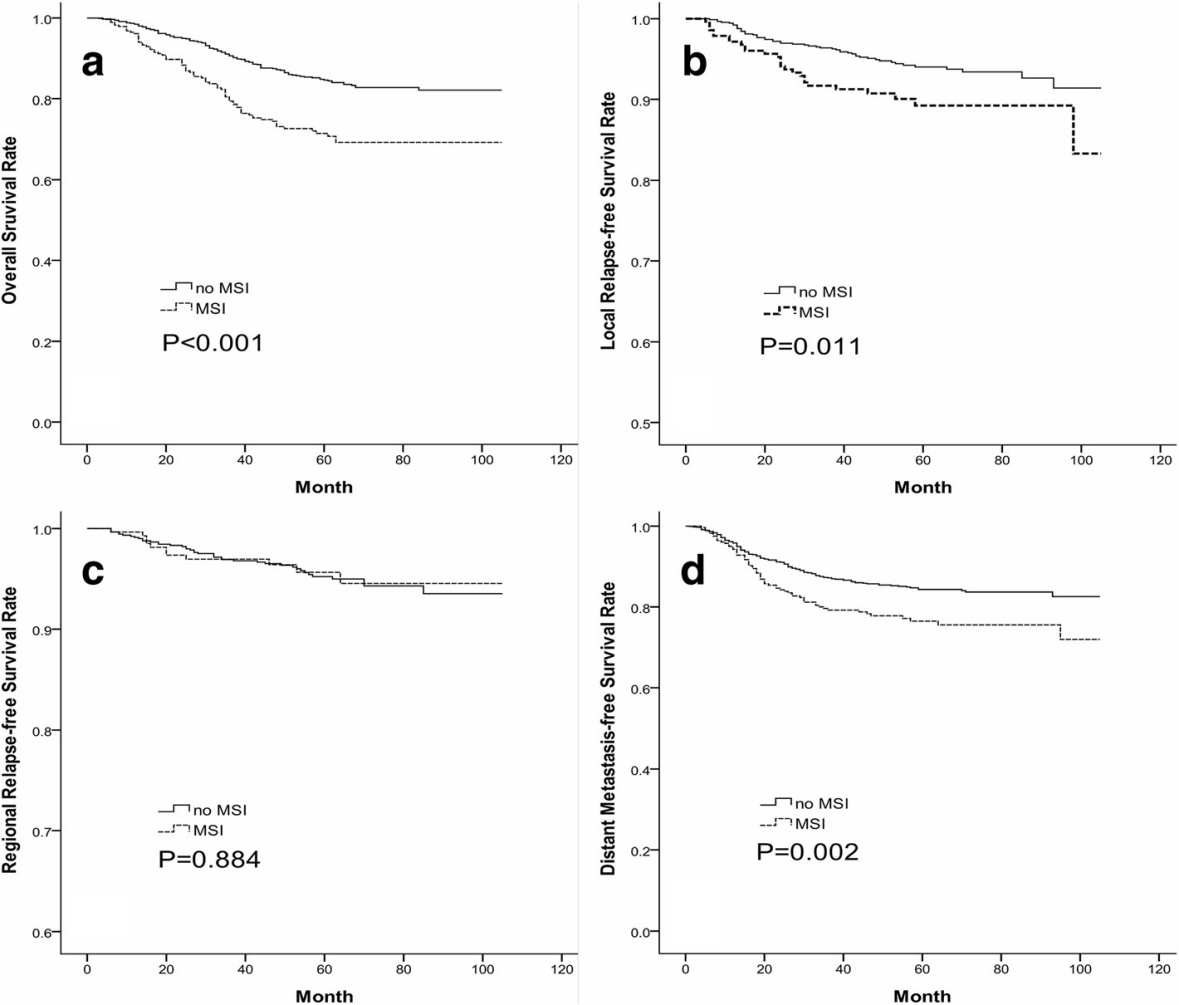
Survival curves compared between NPC patients with and without MSI. **a** Overall survival rate; **b** Local relapse-free survival rate; **c** Regional relapse-free survival rate; **d** Distant metastasis-free survival rate. (NPC = Nasopharyngeal carcinoma; MSI = Masticator space involvement)

**Fig. 3:**
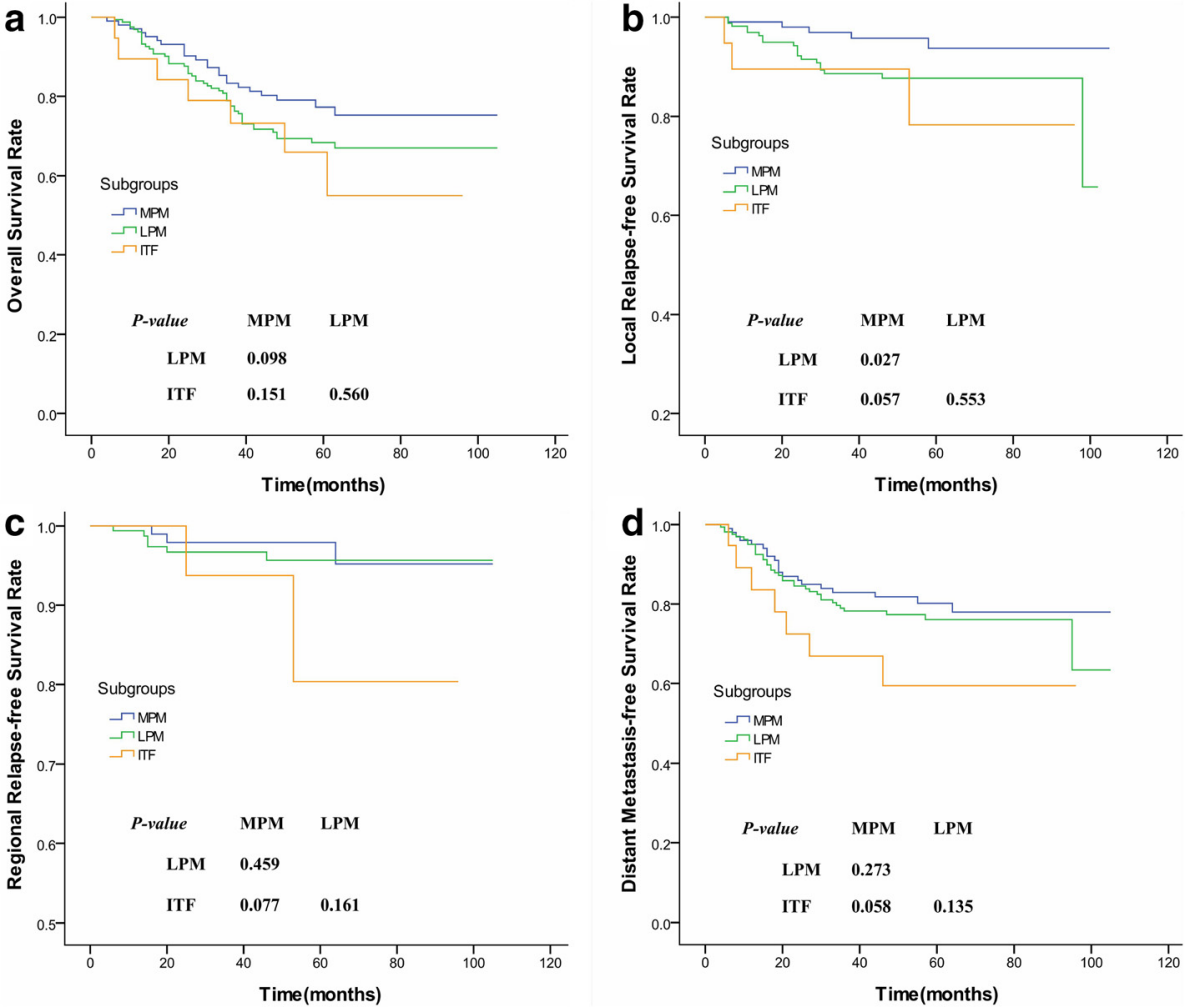
Survival curves compared between subgroups of NPC patients suffering from different degrees of MSI. **a** Overall survival rate; **b** Local relapse-free survival rate; **c** Regional relapse-free survival rate; **d** Distant metastasis-free survival rate. (NPC = Nasopharyngeal carcinoma; MSI = Masticator space involvement; MPM = Medial pterygoid muscle; LPM = Lateral pterygoid muscle; ITF = infratemporal fossa)

The log-rank test showed that the survival outcomes of patients with MSI were significantly worse than those without involvement with regard to OS (71.7 *vs.* 84.8 %; *P* < 0.001), LRFS (90.5 *vs.* 94.3 %; *P* = 0.011), and DMFS (77.7 *vs.* 84.9 %; *P* = 0.002) but not for RRFS (96.1 *vs.* 95.5 %; *P* = 0.884) (Fig. 2). Besides, patients with tumors involving the MPM only presented a higher LRFS than those concurrently affecting the LPM (95.1 *vs.* 87.8 %; *P* = 0.027), even though they had a consistent OS (77.5 *vs.* 68.5 %; *P* = 0.098), RRFS (97.1 *vs.* 95.6 %; *P* = 0.459), and DMFS (80.4 *vs.* 76.2 %; *P* = 0.273; Fig. 3). Patients with ITF erosion behaved a consistent OS, LRFS, RRFS, and DMFS with those involving the MPM and/or LPM (*P* > 0.05; Table 2 and Fig. 3).

**Table 2:** Comparison of survival outcomes between different degrees of masticatory space involvement and T-categories

Log rank (Mantel-Cox)	*P*-value	OS	LRFS	RRFS	DMFS
MPM only *vs.*	T1	0.010	0.114	0.417	0.008
	T2	0.056	0.976	0.333	0.250
	T3	0.434	0.814	0.612	0.742
	T4	0.097	0.008	0.147	0.184
	LPM	0.098	0.027	0.459	0.273
	ITF	0.151	0.057	0.077	0.058
LPM *vs.*	T1	<0.001	<0.001	0.041	<0.001
	T2	<0.001	0.005	0.779	0.007
	T3	<0.001	0.001	0.714	0.057
	T4	0.910	0.482	0.404	0.861
	MPM only	0.098	0.027	0.459	0.273
	ITF	0.560	0.553	0.161	0.135
ITF *vs.*	T1	<0.001	<0.001	0.005	<0.001
	T2	0.004	0.033	0.257	0.004
	T3	0.037	0.034	0.124	0.020
	T4	0.572	0.886	0.412	0.210
	MPM only	0.151	0.057	0.077	0.058
	LPM	0.560	0.553	0.161	0.135

*MPM* medial pterygoid muscle, *LPM* lateral pterygoid muscle, *ITF* infra temporal fossa, OS overall survival, *LRFS* local relapse-free survival, *RRFS* regional relapse-free survival, *DMFS* Distant metastasis-free survival

### The prognosis of patients with masticator space involvement and different T-staging categories

**Fig. 4:**
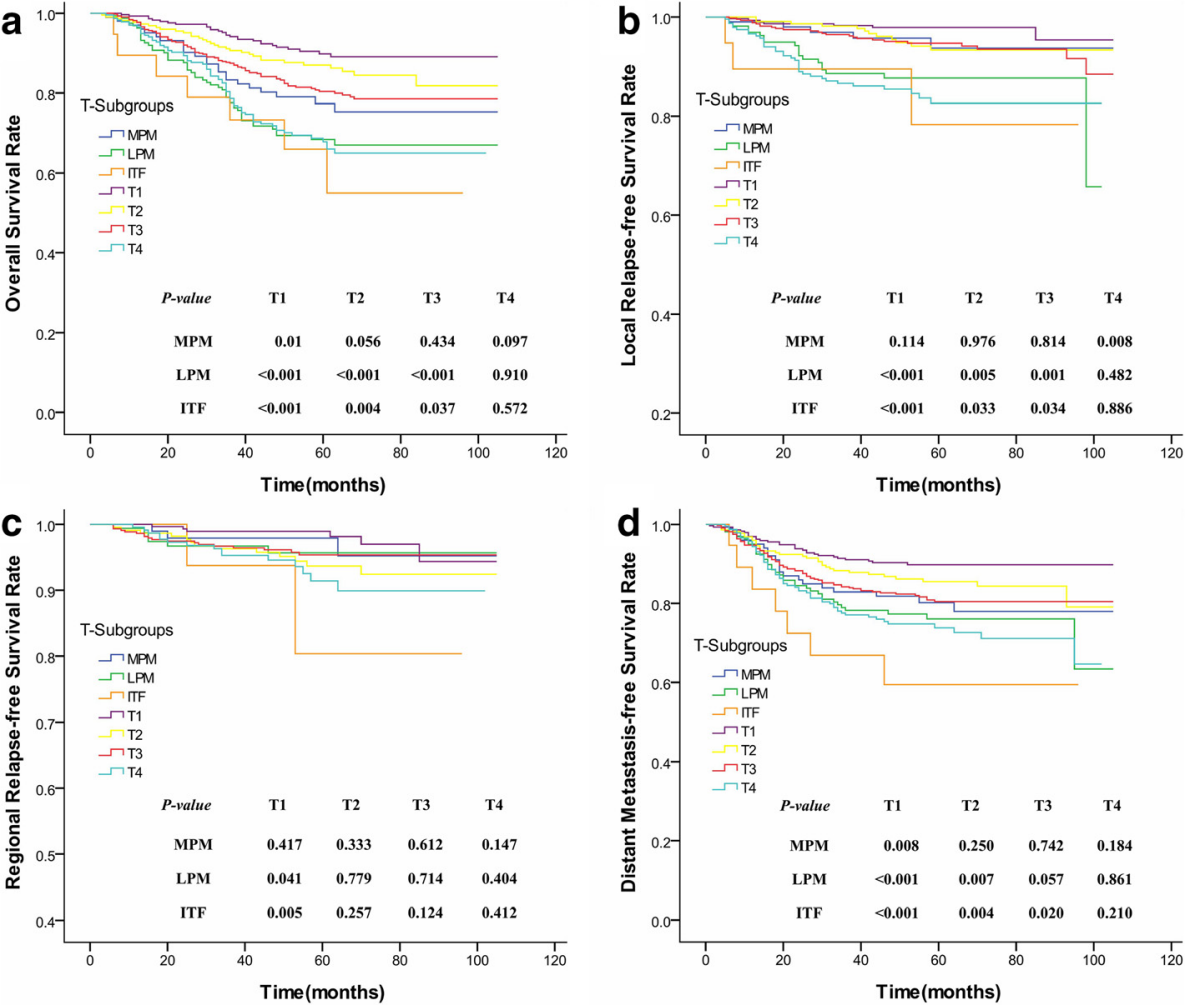
Survival curves compared between subgroups of NPC patients suffering from different degrees of MSI and different T-stage categories. **a** Overall survival rate; **b** Local relapse-free survival rate; **c** Regional relapse-free survival rate; **d** Distant metastasis-free survival rate. (NPC = Nasopharyngeal carcinoma; MSI = Masticator space involvement; MPM = Medial pterygoid muscle; LPM = Lateral pterygoid muscle; ITF = infratemporal fossa; OS = Overall survival; LRFS = Local relapse-free survival; DMFS = Distant metastasis-free survival; RRFS = Regional relapse-free survival)

**Fig. 5:**
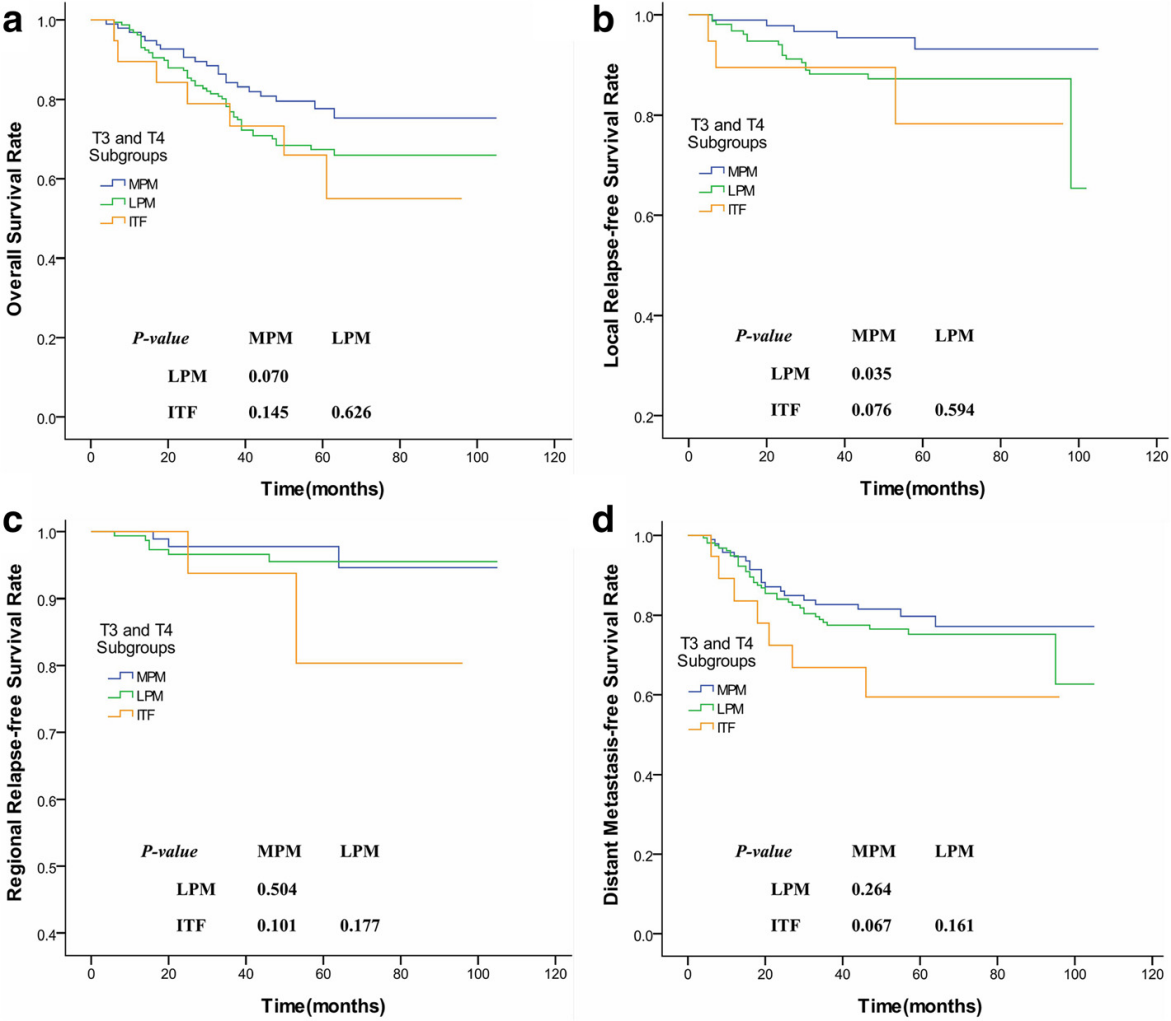
Survival curves compared between different degrees of MSI among NPC patients composed of T3 and T4 stages. **a** Overall survival rate; **b** Local relapse-free survival rate; **c** Regional relapse-free survival rate; **d** Distant metastasis-free survival rate. (MSI = Masticator space involvement; NPC = Nasopharyngeal carcinoma; MPM = Medial pterygoid muscle; LPM = Lateral pterygoid muscle; ITF = infratemporal fossa; NPC = Nasopharyngeal carcinoma)

As for the subgroup of patients with tumors involving the MPM only, their 5-year OS and DMFS rates were significantly worse than the rates of those classified as T1 stage (*P* = 0.01; *P* = 0.008) and were more consistent with a T3 or T4 stage tumor (*P* > 0.05), while the 5-year LRFS rate was not significantly different from those classified as a T2 or T3 disease (*P* > 0.1) and higher than those at T4 stage (*P* = 0.008, Fig. 4). When the tumor concurrently invaded into the LPM or ITF, however, patients had worse 5-year OS and LRFS rates than those classified as stage T1, T2, or T3 (*P* < 0.05). These rates were more consistent with a T4 disease (*P* > 0.1). Moreover, their 5-year RRFS rates were more consistent with those classified as T2, T3, or T4 stage (*P* > 0.1), and their 5-year DMFS rates were more consistent with stage T4 (*P* > 0.1; Fig. 4; Table 2). Nevertheless, among patients classified as a T2, T3 or T4 stage disease, respectively, MSI did not significantly affect their OS, LRFS, RRFS, and DMFS (*P* > 0.05). But for the subgroup of patients composed of T3 and T4 stages, tumors involving the MPM only presented a higher LRFS than those concurrently affecting the LPM (*P* = 0.035; Fig. 5) even though they had a similar OS, RRFS, and DMFS (*P* = 0.070, *P* = 0.504, and *P* = 0.264; respectively). Whereas, patients with the concurrent erosion of ITF remained a consistent OS, LRFS, RRFS, and DMFS with those infiltrating the MPM and/or LPM (*P* > 0.05; Fig. 5).

In the general group, most patients with MSI concurrently involved structures that earned T3 and T4 classifications (271/283, 95.76 %) while only a small proportion of cases without MSI did (406/914, 44.42 %). In the subgroup of patients with different degrees of MSI, a majority of tumors involving the MPM only had concurrent erosions of T3-stage structures, while most of those with LPM involvement concurrently infiltrated T4-stage structures, and 100 % of subgroup patients with ITF erosion affected other T4-stage structures concurrently (see Table 3). Table 3 lists the frequencies of concurrent erosions at other sites between different degrees of MSI.

**Table 3:** Frequencies of concurrently involving sites for NPCs with different degrees of MSI

Concurrent involvement sites	no MSI	MSI	MPM only	LPM	ITF
No. of patients	914	283	102	181	19
Nasal fossa	106 (11.60 %)^a^	75 (26.50 %)	25 (24.51 %)	50 (27.62 %)	6 (31.58 %)
Pre-vertebral muscles	171 (18.71 %)	126 (44.52 %)	36 (35.29 %)	90 (49.72 %)	11 (57.89 %)
Oropharynx	2 (0.22 %)	19 (6.71 %)	4 (3.92 %)	15 (8.29 %)	1 (5.26 %)
Pterygopalatine fossa	96 (10.50 %)	115 (40.64 %)	26 (25.49 %)	89 (49.17 %)	15 (78.95 %)
Paranasal sinuses	76 (8.32 %)	90 (31.80 %)	20 (19.61 %)	70 (38.67 %)	10 (52.63 %)
Skull base bones	387 (42.34 %)	267 (94.35 %)	93 (91.17 %)	174 (96.13 %)	19 (100.00 %)
Oval foramen	55 (6.02 %)	153 (54.06 %)	43 (42.16 %)	110 (60.77 %)	12 (63.16 %)
Foramen	25 (13.13 %)	72 (25.44 %)	12 (11.76 %)	60 (33.15 %)	12 (63.16 %)
Orbit	4 (0.44 %)	11 (3.89 %)	1 (0.98 %)	10 (8.39 %)	2 (10.53 %)
Cranial nerves	33 (3.61 %)	58 (20.49 %)	7 (6.86 %)	51 (28.18 %)	9 (47.37 %)
Cavernous sinus	63 (6.89 %)	109 (38.52 %)	21 (20.59 %)	88 (48.61 %)	8 (42.11 %)
Meninges	47 (5.14 %)	107 (37.81 %)	26 (24.49 %)	81 (44.75 %)	10 (52.63 %)

*NPC* nasopharyngeal carcinoma, *MSI* masticaor space involvement, *MPM* medial pterygoid muscle, *LPM* lateral pterygoid muscle, *ITF* infratemporal fossa

^a^Numbers in parentheses are percentages

The frequencies of concurrent erosion at other T3 or T4-stage structures in the subgroup of patients with MPM involvement were mostly lower than in the subgroup of LPM extension, particularly at the pterygopalatine fossa (25.49 *vs.* 49.17 %, *P* < 0.05), para-nasal sinus (19.61 *vs.* 38.67 %, *P* < 0.05), cavernous sinus (20.59 *vs.* 48.61 %, *P* < 0.05), foramen (11.76 *vs.* 33.15 %, *P* < 0.05), oval foramen (42.16 *vs.* 60.77 %, *P* < 0.05), meninges (24.49 *vs.* 44.75 %, *P* < 0.05) and cranial nerves (6.86 *vs.* 27.07 %, *P* < 0.05). And the majority of these above frequencies were relatively lower than in patients with ITF invasion (see Table 3).

### Multivariate analyses of prognostic factors

Cox regression analysis was employed by multivariate analyses to systematically evaluate how various prognostic factors affected patients’ survival outcomes. The following factors were taken into the calculation in the Cox regression analysis: gender, histopathology, age (≥50 years *vs.* <50 years), T classification (T1-2 *vs.* T3-4), N classification (N0-1 *vs.* N2-3), clinical staging (Stage I, II *vs.* III, IV), parapharygeal space extension, skull base erosion, paranasal sinus erosion, degrees of MSI (no MSI *vs.* MPM only *vs.* LPM *vs.* ITF), pterygopalatine fossa infiltration, perineural spread, intracranial invasion, and chemotherapy. As a result, degrees of MSI were demonstrated to be an independent prognostic factor for the OS significantly (*P* = 0.036) but not for the LRFS (*P* = 0.526), RRFS (*P* = 0.539), and DMFS (*P* = 0.401), respectively. Other insignificant prognostic factors for the OS, LRFS, RRFS, and DMFS included the variables of gender, histopathology, parapharygeal space extension, skull base erosion, paranasal sinus erosion (*P* > 0.05). On the other hand, independent prognostic factors for the OS included age, N classification, pterygopalatine fossa infiltration, perineural spread, and intracranial invasion (*P* < 0.05). For the LRFS, the independent factors were age, N classification, pterygopalatine fossa infiltration, perineural spread, and intracranial invasion (*P* < 0.05). Only N classification and perineural spread were demonstrated as independent factors for the RRFS (*P* < 0.05), while the independent factors for the DMFS were identified as age, T classification, N classification, perineural spread, and chemotherapy (*P* < 0.05, see Table 4).

**Table 4:** Multivariate analysis of prognostic factors in IMRT-treated NPC patients

Endpoint	Variable	*P-value*	Odds ratio^a^
Death	N classification (N0–1 *vs*. N2–3)	<0.001	1.569 (1.281, 1.922)
	Age (≥50 y *vs.* < 50 y)	<0.001	2.653 (2.012, 3.498)
	Pterygopalatine fossa infiltration	0.005	0.555 (0.368, 0.837)
	Degrees of MSI (no MSI *vs.* MPM only *vs.* LPM *vs.* ITF)	0.036	1.233 (1.014, 1.499)
	Intracranial invasion	0.010	22.052 (1.860, 261.444)
	Perineural spread	0.004	1.671 (1.073, 2.600)
Local failure	T classification (T1–2 *vs.* T3–4)	0.248	1.301 (0.833, 2.034)
	N classification (N0–1 *vs*. N2–3)	0.033	1.437 (1.031, 2.004)
	Age (≥50 y *vs.* < 50 y)	0.036	1.613 (1.030, 2.525)
	Pterygopalatine fossa infiltration	0.017	1.961 (1.130, 3.405)
	Degrees of MSI (no MSI *vs.* MPM only *vs.* LPM *vs.* ITF)	0.526	0.906 (0.668, 1.229)
	Intracranial invasion	0.020	32.712 (3.459, 309.393)
	Perineural spread	0.006	2.474 (1.302, 4.699)
Regional failure	N classification (N0–1 *vs*. N2–3)	0.015	1.752 (1.117, 2.746)
	Degrees of MSI (no MSI *vs.* MPM only *vs.* LPM *vs.* ITF)	0.539	0.873 (0.566, 1.347)
	Perineural spread	0.049	2.477 (1.004, 6.112)
Distant failure	T classification (T1–2 *vs.* T3–4)	0.037	1.431 (1.022, 2.004)
	N classification (N0–1 *vs.* N2–3)	<0.001	1.769 (1.412, 2.217)
	Age (≥50 y *vs.* < 50 y)	0.014	1.431 (1.074, 1.906)
	Perineural spread	0.012	1.871 (1.150, 3.042)
	Degrees of MSI (no MSI *vs.* MPM only *vs.* LPM *vs.* ITF)	0.401	1.093 (0.888, 1.345)
	Chemotherapy	0.048	1.838 (1.006, 3.359)

*NPC* nasopharyngeal carcinoma, *IMRT* intensity modulated radiation therapy, *MSI* masticator space involvement, *MPM* medial pterygoid muscle, *LPM* lateral pterygoid muscle, *ITF* infratemporal fossa

^a^Numbers in parentheses give 95 % confidence intervals

## Discussion

Our present results indicate that NPCs with MSI had a worse OS, LRFS, and DMFS than those without involvement. Even that the degree of MSI was not a significant prognostic factor for the LRFS, RRFS and MDFS, it was demonstrated as the independent prognostic factor for patients’ overall survival rates. Furthermore, the subgroup of patients with an erosion of the lateral masticator space (i.e., LPM and ITF) presented a poorer prognosis (more consistent with T4 stage) than those affecting the MPM only (consistent with T3 stage). Thus, NPC with the lateral MSI extension was recommended to be classified as a higher T-stage category.

Anatomical MS is enclosed by the superficial layer of the deep cervical fascia, and this space contains not only the masticatory muscles but also the mandible and the cranial nerve of V3 [23, 24]. Thus, imaging modalities (i.e., CT, MRI) are always employed to characterize the complex anatomical features of MS. In particular, MRI is superior to CT in the diagnosis of MSI, with a higher tissue contrast and sensitivity, which allows us to stage a large number of NPC patients more efficiently. It is interesting that MRI diagnosed a higher frequency of MSI in our present study than in previous studies conducted by Tang et al. [9] (23.64 *vs.* 19.7 %) and Zhang et al. [11] (23.64 *vs.* 20.2 %), even for the subgroups of tumor involving the MPM only (23.64 *vs.*18.70 %; 23.64 *vs.* 20.00 %; respectively) and the LPM (15.12 *vs.* 10.00 %; 15.12 *vs.* 8.40 %; respectively). In another previous study, Sze et al. [10] observed a much higher frequency for both MSI (61.00 %) and the erosion of MPM only (43.84 %), however, that of LPM invasion was relatively lower at 11.96 %. Moreover, all patients with MSI in this study had tumors invading the MPM, and any tumor with erosion of LPM or ITF also concurrently affected the MPM, which is quite different from the previous studies [9–11].

In the staging of NPC, MSI has always been taken as a key indicator for stage T4 classification, which reflects the observed risk for distant metastasis and tumor recurrence in patients treated for non-metastatic NPC [9, 25]. When surveyed, however, the prognostic value of MSI varies considerably among different previous studies. Tang et al.’s study suggested MSI predicted survival outcomes that were consistent with a T4 disease and argued that the levels of MSI did not correlate with patient’s OS and LRFS [9]. In contrast, Sun et al. [26] and Zhang et al. [11] both claimed different patterns of MSI presented distinct survival outcomes, and the LPM erosion correlated with a T4 stage while the involvement of only MPM correlated with a T2 stage [11], which agrees with our results. On the other hand, Chen et al. demonstrated that in the subgroup of patients with the T4 stage, tumors involving the MS (T4a) presented a higher OS and DMFS rates than those involving other T4 structures (T4b), while their LRFS rates were not significantly different [25]. Pan et al.’s results also suggest a subset of patients with MSI should be up-staged to T3 or T4 even though the involvement of MPM and LPM was not associated with the poor prognosis linked to other structures [12].

MSI has been regarded as a significant prognostic factor for the prognosis of NPCs [9], and under most circumstances the extent of involvement strictly correlated with the survival outcomes we measured. Our present results also showed that MSI, especially the lateral MSI extension, did bring a relatively poorer survival outcome and the degree of MSI was demonstrated as an independent prognostic factor for the OS even though it was not for the LRFS, RRFS and DMFS. In addition, tumors with lateral MSI extension always came with concurrent involvement at other sites that, on their own, could stage the tumors as T3 or T4 classification. (These regions associate with T4 tumors include the pterygopalatine fossa, V2 or V3, or orbit and are independent factors for a poor prognosis.) As shown in Table 3, frequencies of concurrent erosion of other T3 or T4 structures were higher in patients with MSI than in those without MSI. Thus, the higher rates of concurrently involving the further T-stage structures may result in worse survival outcomes for patients with MSI. Moreover, in this study, only a few patients were finally proven to have MSI alone (no involvement of other T3 or T4 structures as well), and these patients always displayed a relatively better survival outcome which was consistent with the T1 or T2 stages, which agrees with Sze et al.’s study [10]. On the other hand, even though whether or not the tumor involved the MPM or LPM had no effects on the patients’ OS and DMFS, tumors involving the MPM only were linked to a higher LRFS than those infiltrating the LPM. However, NPCs involving the LPM (as opposed to the MPM) always tended to more frequently affect other sites of the T4 stage, which always linked to an aggressive disease and poor prognosis (Table 3). As for patients classified as different T-staging categories, those with tumor erosions of LPM or ITF used to affect concurrently other structures that elicited an equivalent or higher T stage. In this sense, when evaluating the prognostic value of MSI in NPC patients, these sites of concurrent involvement should be weighted and balanced heavily.

Generally, NPC originates from the nasopharyngeal mucosa, in a stepwise fashion, moving laterally into the parapharyngeal space and then spreading directly into the anatomical MS. Moreover, tumors invading the pterygopalatine fossa also find a route to spread into masticator space indirectly. Therefore, among patients with MSI, all of them had concurrent involvement in the parapharyngeal space, and some even displayed concurrent erosion of the pterygopalatine fossa with a percentage of 25.49 *vs.* 49.17 % for MPM only *vs.* LPM (*P* < 0.05). Furthermore, it was reported that a tumor’s extension to parapharyngeal space, pterygopalatine fossa, and masticator space was always associated with a higher risk of distant metastasis and tumor recurrence [26–28]. Tang et al. suggested MSI would increase the risk of distant metastasis and tumor recurrence and should be categorized as a T4 tumor [9]. However, these studies did not evaluate the importance of MSI itself, so the negative impacts on the OS, LRFS and DMFS (*P* < 0.05) can be attributed to the fact that tumors that spread to the MS generally also affect other structures associated with more aggressive diseases. Sze et al. [10] believed that NPC patients with MSI alone displayed a better survival outcome, which agrees with this study. Since the pterygopalatine fossa contains the maxillary division of the trigeminal nerve (V2), a tumor’s spread into this region should be expected to increase the risk of V2 infiltration and worsen the survival outcomes. In this study, the frequency of tumors concurrently infiltrating V2 among subgroup patients with the involvement of MPM only *vs.* LPM was 11.76 *vs.* 33.15 %, respectively. Similarly, the mandibular division of the trigeminal nerve (V3) runs through the interval space lying between the MPM and LPM. When tumors concurrently involved the LPM, the risk for the infiltration of V3 would dramatically increase. In the present results, patients with the LPM erosion owned a higher rates of concurrent V3 infiltration than those with MPM involvement (60.77 *vs.* 42.16 %; *P* < 0.05). Therefore, these perineural and intracranial spreads that were demonstrated as significant prognostic factors in the multivariate analysis would primarily result in a poorer LRFS for patients with LPM involvement. Nevertheless, thanks to the ever-improving local control rate afforded by IMRT, outcomes are improving even for patients harboring aggressive tumors.

Even though MSI is known to worsen NPC’s survival outcomes, its T-staging categories are always inconsistent and controversial [9–13]. It is observed that NPC patients with the involvement of MPM only showed a consistent OS and LRFS with a T2 or T3 diagnosis whereas those concurrently affected the LPM were more consistent with a T4 disease. However, only 5/225 (2.22 %) of T2 cases and 56/441 (12.70 %) of T3 cases had tumors infiltrating the LPM while the frequency was much higher for T4 cases at 120/236 (50.84 %) (Table 1). Thus, NPCs involving the LPM more often concurrently spread into the deeper structures (i.e., pterygopalatine fossa, cranial nerves, and intracranial structures), which obviously worsen the survival outcomes. Chen et al. [25] asserted NPC patients categorized as the T4a subclassification (with involvement of MS) had a significantly higher OS (*P* = 0.033) and DMFS (*P* = 0.036) than those of the T4b subclassification (without involvement of MS). Sze et al. also indicated that NPCs infiltrating the MPM and/or LPM alone (without invasion to other T3 and T4 structures) always owned a better survival outcome and should be staged as T2 diseases [10]. (That is unless the tumor has invaded structures that, in their own right, render a classification of T3 or T4.) Our present results agree with these assertions.

Among MSI patients, only 12 (4.24 %) cases involved the MS alone (without involvement of any T3 or T4 structures), including 7 cases that invaded the MPM only and other 5 that affected both the MPM and LPM, yielding a much smaller proportion of only 1 % among the entire group of NPC patients. Tang et al. reported a similar lower frequency of MSI alone (2.2 %) [9] that agrees with the present results. However, those patients with MSI alone were clearly demonstrated to diaplay a dramatically better survival outcome among which no local or regional recurrence was observed by the ultimate follow-up. Therefore, NPC tumors with MSI alone may be more reasonable to classify as a T2-stage as was suggested by Sze et al. [10]. In contrast, 5-year OS, LRFS, and DMFS rates of patients with erosions of ITF were relatively poorer at 63.16, 84.21, and 63.16 % respectively, and different editions of the AJCC staging system have identified the ITF as the lateral part of anatomical MS and thus, categorised a tumor’s spread into this region as a T4 disease [13]. Even though they were not significantly different from those with MPM and/or LPM infiltration, the frequencies of temporal and masseter muscle abnormalities were both much lower in the entire group (1.09 and 0.33 %, respectively) and may be insufficient in statistical analysis, which also agrees with Tang et al.’s study [9] Furthermore, a tumor’s lateral extension into ITF always tended to concurrently affect other sites that rendered a T4 classification (i.e., the cavernous sinus, cranial nerves, and meninges). Therefore, the lateral extension of MSI (i.e., LPM and ITF) might sharply worsen patient’s survival outcomes and should be classified as higher T-stage categories as was recommended by the 2008 Chinese staging system.

The limitation of this present study is that for such a retrospective study, the sample size was relatively insufficient, so another study with a larger sample size is needed. Furthermore, this study was conducted with data from a single medical center. Thus, a prospective study of multiple centers should derive a more unbiased result.

## Conclusions

Multivariate analyses indicate that the degree of MSI is a significant prognostic factor for the OS of NPCs treated with IMRT, NPCs with tumors infiltrating the LPM and ITF present poorer prognostic outcomes than those affected the MPM only and T3-stage disease, and those with MSI alone always associate with relatively higher survival rates. Thus, it is highly recommended that tumors with MSI alone be classified under the T2-stage while the lateral MSI extension (LPM and ITF) be classified as higher T-staging categories in the next revision of T-staging for NPCs.

## Abbreviations

NPC: Nasopharyngeal carcinoma
IMRT: Intensity-modulated radiation therapy
MRI: Magnetic resonance imaging
MS: Masticator space
MSI: Masticator space involvement
MPM: Medial pterygoid muscle
LPM: Lateral pterygoid muscle
ITF: Infratemporal fossa
AJCC: American Joint Committee on Cancer
OS: Overall survival
LRFS: Local relapse-free survival
RRFS: Regional relapse-free survival
DMFS: Distant metastasis-free survival
